# Metabarcoding of Parasitic Wasp, *Dolichogenidea metesae* (Nixon) (Hymenoptera: Braconidae) That Parasitizing Bagworm, *Metisa plana* Walker (Lepidoptera: Psychidae)

**DOI:** 10.21315/tlsr2022.33.1.2

**Published:** 2022-03-31

**Authors:** Aqilah Sakinah Badrulisham, Muhammad Abdul-Latiff Abu Bakar, Badrul-Munir Md. Zain, Shukor Md-Nor, Mohd-Ridwan Abd Rahman, Nur Syafika Mohd-Yusof, Madihah Halim, Salmah Yaakop

**Affiliations:** 1 Centre for Insect Systematics, Department of Biological Science and Biotechnology, Faculty of Science and Technology, Universiti Kebangsaan Malaysia, 43600 Bangi, Selangor, Malaysia; 2 Department of Biological Science and Biotechnology, Faculty of Science and Technology, Universiti Kebangsaan Malaysia, 43600 Bangi, Selangor, Malaysia; 3 Centre of Research for Sustainable Uses of Natural Resources (CoR-SUNR), Faculty of Applied Sciences and Technology, Universiti Tun Hussein Onn Malaysia (Pagoh Campus), 84600, Muar, Johor, Malaysia; 4 Centre for Pre-University Studies, Universiti Malaysia Sarawak, 94300 Kota Samarahan, Sarawak, Malaysia

**Keywords:** Endoparasitoids, Endosymbiont, Microbiome, Parasitic Wasp, Malaysia, Endoparasitoid, Endosimbion, Mikrobiom, Penyengat Parasitoid, Malaysia

## Abstract

Microbiome studies of the parasitoid wasp, *Dolichogenidea metesae* (Nixon) (Hymenoptera, Braconidae) are important because *D. metesae* has potential as a biological control agent to suppress the pest, *Metisa plana* Walker (Lepidoptera, Psychidae). Three field populations of parasitic wasps with different Integrated Pest Management (IPM) practices to control *M. plana* collected from Perak state (Tapah) and Johor state (Yong Peng and Batu Pahat districts) in Peninsular Malaysia were studied. Bacterial community composition and structure were analysed using α and β diversity metrics. Proteobacteria (83.31%) and Bacteroidetes (6.80%) were the most dominant phyla, whereas unknown family from order Rhizobiales was the most abundant family found in all populations followed by Pseudomonadaceae. Family Micrococcaceae was absent in Tapah. Rhizobiales gen. sp. and *Pseudomonas* sp. were abundant in all populations. Pearson’s correlation analysis showed the strongest correlation between individuals of Batu Pahat and Yong Peng (*r* = 0.89827, *p* < 0.05), followed by Tapah and Yong Peng with *r* = 0.75358, *p* < 0.05 and Batu Pahat and Tapah (*r* = 0.69552, *p* < 0.05). We hypothesise that low diversity and richness in Tapah might be due to direct and indirect effect of insecticides application. This preliminary data was the first study to do inventory of the microbiomes in the gut of the *D. metesae*.

## Abstract (fr)

Kajian mikrobiom ke atas spesies penyengat parasitoid, *Dolichogenidea metesae* (Nixon) (Hymenoptera, Braconidae) adalah penting kerana spesies ini berpotensi sebagai agen kawalan biologi untuk mengawal spesies perosak, *Metisa plana* Walker (Lepidoptera, Psychidae). Tiga populasi penyengat parasitoid yang mengaplikasikan Pengurusan Perosak Bersepadu (IPM) yang berbeza untuk mengawal *M. plana* iaitu daripada negeri Perak (Tapah) dan negeri Johor (Yong Peng dan Batu Pahat) di Semenanjung Malaysia telah dikaji. Komposisi dan struktur komuniti bakteria telah dianalisis menggunakan metrik kepelbagaian α dan β. Proteobacteria (83.31%) dan Bacteroidetes (6.80%) merupakan filum yang paling dominan, manakala famili yang tidak diketahui daripada order Rhizobiales ialah famili yang paling melimpah ditemui dalam kesemua populasi diikuti oleh Pseudomonadaceae. Famili Micrococcaceae didapati tidak hadir di populasi Tapah. *Rhizobiales* gen. sp. dan *Pesudomonas* sp. adalah paling melimpah di dalam semua populasi. Analisis korelasi Pearson menunjukkan korelasi yang paling tinggi di antara individu daripada Batu Pahat dan Yong Peng (*r* = 0.89827, *p* < 0.05) diikuti oleh Tapah dan Yong Peng dengan nilai *r* = 0.75358, *p* < 0.05 dan Batu Pahat dan Tapah (*r* = 0.69552, *p* < 0.05). Kami menghipotesis bahawa kepelbagaian dan kelimpahan yang rendah di Tapah mungkin disebabkan oleh kesan langsung dan tidak langsung penggunaan racun serangga. Data awalan ini merupakan kajian pertama yang melakukan inventori mikrobiom ke atas usus *D. metesae*.

**Highlight**

- First inventory of microbiome presents in the gut of parasitic wasp from three different populations in Peninsular Malaysia.
- Dominant phylum, families and genera were recorded from the three populations.
- The diversity and richness of microbial communities in the gut of *D. metesae* were hypothesised to be affected by the direct and indirect usage of insecticides.

## INTRODUCTION

Parasitoids are those natural enemies that are multicellular (in contrast to pathogens) and directly cause death of their host (in contrast to parasites) (Haelewaters *et al.* 2017). Parasitoids play an important role as biological control agents in suppressing populations of bagworm (Lepidoptera, Psychidae) (Cheong *et al.* 2010; Hanysyam *et al.* 2013; Kamarudin *et al.* 2017). The bagworm species *Metisa plana* Walker is the dominant pest infesting palm oil plantations in Peninsular Malaysia (Kamarudin *et al.* 2019). Consequences of infestations are becoming increasingly serious (Kamarudin & Arshad 2016). Hence, chemical insecticides (Kok *et al.* 2012), biopesticides (Kamarudin *et al.* 2010; Mazmira *et al.* 2011), and pheromone applications (Kamarudin *et al.* 2019) have been used in controlling *M. plana*. In addition, natural enemies have been used to reduce populations of bagworm (Basri *et al.* 1995; Ali *et al.* 2007).

Several studies have been conducted on the diversity, ecology and insecticides resistance of the parasitoids that use *M. plana* Walker as their host (Kamarudin *et al.* 1996; Hanysyam *et al.* 2013; Potineni & Saravanan 2013; Halim, Aman-Zuki, *et al.* 2018; Halim, Din, *et al.* 2018). The wasp *Dolichogenidea metesae* (Nixon) (Hymenoptera, Braconidae) is known as the most effective parasitoid species against *M. plana* (Halim, Aman-Zuki, *et al.* 2018). As a result, this species has high potential to be commercialised as a biological control agent in the oil palm industry. Still, genetic variation needs to be considered on *D. metesae* prior as part of risk assessment programmes. This is to ensure the effectiveness of the parasitoid as a biological control agent when released to the field (Paspati *et al.* 2019). For mass rearing purposes, we must select the most genetically diverse population (Freitas *et al.* 2018).

Interestingly, *D. metesae* lives in the body of its host, *M. plana*. As endoparasitoids, the wasp lives in the host body from egg to the adult stage, receiving nutrients from the host while slowly killing it (Harvey & Malcicka 2016). Many papers are published on the interaction between these two species, e.g., Halim, Din, *et al.* (2018), but no information is available on the gut microbiome of *D. metesae*. This is in part due to the low number of bacteria that can be cultured (Dudek *et al.* 2017). A good understanding of the microbial communities, including endosymbionts, will help to determine differences among populations of this parasitoid wasp (Werren *et al.* 2008; Whitfield 2016).

Microbial data is crucial to understand the associations of the insects and microorganisms that can be either pathogenic or symbiotic (Sanchez-Contreras & Vlisidou 2008; Douglas 2015). On the other hand, these associations also contribute to micro-evolutionary processes in the insects, leading to diversification (Sanchez-Contreras & Vlisidou 2008). The symbiotic interactions with bacteria mainly developed within the insect gut (Hooper 2001); some insect species rely on their symbiotic microorganisms for enhanced food digestion, communication, nutrition or defense (Cardoza *et al.* 2012; Engel & Moran 2013).

Metagenomics is the direct genetic analysis of genomes contained in an environmental sample (Thomas *et al.* 2012). It offers a path to the study of community structures, phylogenetic composition, species diversity, metabolic capacity and functional diversity of microbes (Shah *et al.* 2011). Yet, there are only a few studies on insects using metagenomics analysis focusing on gut bacterial diversity (Yun *et al.* 2014; Nedoluzhko *et al.* 2017). Here, we propose to use microbiome data to investigate the role of microbes in the potential application of *D. metesae* as an alternative biological control agent of *M. plana*. This study evaluated the diversity of the bacterial communities that are present in the gut of *D. metesae* from three locations with different Integrated Pest Management (IPM) practices. A metagenomics analysis was implemented to sequence the microbial species using the 16S ribosomal RNA (rRNA) gene.

## MATERIALS AND METHODS

### Sample Collection

*D. metesae* individuals were obtained from reared bagworm species, *M. plana* in the laboratory. The parasitized bagworms were collected from infested oil palm plantations in Batu Pahat (Johor) (1°58′45.0″N 102°57′25.0″E), Yong Peng (Johor) (2°08′37.8″N 103°02′23.3″E) and Tapah (Perak) (4°09′23.2″N 101°16′22.5″E) of Peninsular Malaysia, which have different methods for controlling the *M. plana*, namely natural control, biopesticides (*Bacillus thuringiensis*) and chemicals application, respectively (Table 1). A total of six samples of female *D. metesae* were used in this study representing the three sampling sites because only female species act as parasitoids by laying eggs inside host (*M. plana*) (Koppik *et al.* 2019). Dissection of the gut of *D. metesae* was conducted for DNA extraction (Krishnan *et al.* 2014).

### DNA Extraction and Library Preparation

**Figure 1:**
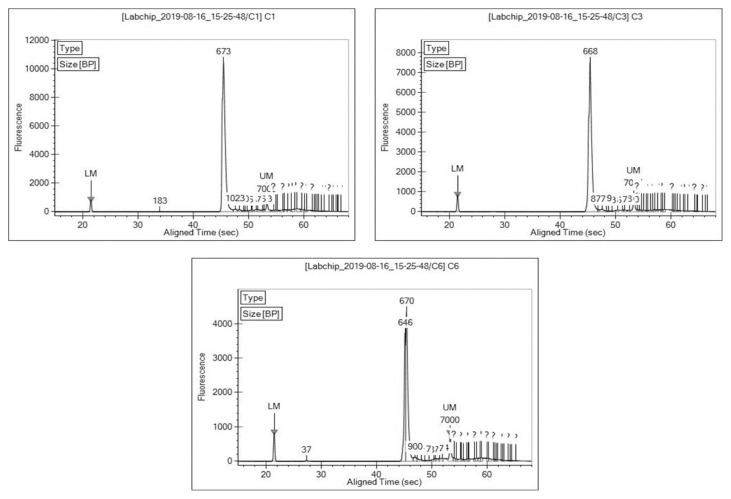
Egrams showing 16S peaks for samples from Batu Pahat (C1), Yong Peng (C3) and Tapah (C6), respectively.

Microbial DNA was extracted from *D. metesae* samples using innuPREP Stool DNA Kit (Analytik Jena, Germany). The evaluation of DNA template quantity and purity was carried out using Implen Nano Photometer and a Qubit 4 Fluorometer (Life Technologies, USA). Two samples of *D. metesae* from each locality were pooled together prior to amplification. A total of 5 uL of each 20 ng/uL of DNA were pooled together and used as DNA for amplification process. Preparation of the library was done through two rounds of Polymerase Chain Reaction (PCR) step. The first PCR was carried out using primers targeting the V3-V4 regions of the 16S rRNA gene which are 16S amplicon PCR forward primer (5′-TCGTCGGCAGCGTCAGATGTGTAT AAGAGACAGCCTACGGGNGGCWGCAG-3′) and 16S amplicon PCR reverse primer (5′-GTCTCGTGGGCTCGGAGATGTGTATA AGAGACAGGACTACHV GGGTATCTAATCC-3′) (Klindworth *et al.* 2013). The first PCR mastermix was composed of 12.5 μL of KAPA HiFi HotStartReadyMix 2X Master Mix (KAPA Biosystems, Wilmington, MA), 5 μL for each forward and reverse primers and 2.5 μL of DNA to a final volume of 25 μL. The amplification was performed on Alpha^TM^PCRmax Alpha Cycler under the following protocols: Initial denaturation of 95°C for 3 min, followed by 35 cycles of denaturation at 95°C for 30 s, primer annealing at 55°C for 30 s, and extension at 72°C for 30 s, with a final elongation at 72°C for 5 min. Amplified PCR products were checked on a 1.5% of agarose gel. The DNA is then purified using 0.7× volume ratio of KAPA pure beads (KAPA Biosystems, USA). Second PCR step for index tagmentation were done using Illumina Nextera XT Index Kit V2 (Illumina Inc, USA). The PCR mixture consist of 5 μL for each Index 1 and Index 2 primer, 12.5 μL of 2× KAPA HiFi HotstartReadyMix, and 2.5 μL of purified DNA to a final volume of 25 μL. PCR amplification for DNA templates with indexes were performed using following profiles: polymerase activation at 72°C for 3 min, initial denaturation at 95°C for 30 s, followed by 12 cycles of denaturation at 95°C for 10 s, annealing at 55°C for 30 s and extension at 72°C for 30 s, and a final extension at 72°C for 5 min. After each step, concentration of DNA for each sample was measured using Qubit 4.0 fluorometer (Qubit dsDNA HS Assay Kit) for quality control purposes. The library was visualised under gel electrophoresis using 1.5% agarose gel in 1× TAE buffer. The size of amplicons of PCR products was analysed using LabChip^®^ GX and the results were visualised in Egram where peak indicating amplicons size can be observed (Fig. 1).

### Quantification and Next Generation Sequencing (NGS)

The library was then quantified using KAPA SYBR^®^ FAST qPCR Master Mix (KAPA Biosystems, USA). The qPCR mixture contains 10 μL KAPA SYBR^®^ FAST qPCR Master Mix; 2 μL of primer premix, 4 μL of indexed-amplicon, and 4 μL of RNase-free distilled water to a final volume of 20 μL. The PCR reaction was carried out on a PCRmax Eco 48 Real Time PCR system under following PCR conditions: initial denaturation at 95°C for 5 min, followed by 25 cycles consisting of denaturation 95°C for 40 s, annealing at 60°C for 2 min, extension at 72°C for 1 min and a final extension step at 72°C for 7 min according to manufacturer’s protocol. PCR products from qPCR were quantified using Qubit 4 Fluorometer for concentration assessment. Normalisation was carried out for each sample of *D. metesae* to ensure even read distribution for all samples. The amplicons were normalised to 4 nM based on data generated by qPCR, LabChip and Qubit quantification. The 5 μL was taken from all 4 nM libraries for library pooling. A total of 500 μL consisting of indexed-amplicons and PhiX were added together for library preparations. PhiX was spiked in together to act as control during sequencing reactions which 40% of the sequencing reads were from PhiX (Jeon *et al.* 2015). Addition of PhiX is important to provide a quality control for cluster generation, sequencing, and alignment, and for calibration control for crosstalk matrix generation, phasing and prephasing. The sequencing length on the Illumina MiniSeq platform is 2× 150 cycles using a MiniSeq High throughput Reagent Kit (Illumina Inc., USA). Sequencing was conducted at Evolutionary and Conservation Genetic Laboratory, Department of Technology and Natural Resources, Universiti Tun Hussein Onn Malaysia (UTHM).

### Bacterial 16S rRNA Gene Sequence Analysis

Reads in FASTQ format were quality-filtered and the adaptors were removed using Genomic Workbench software (CLC) (Qiagen, USA). The OTUs taxonomic classification was against Green genes 16S rRNA database with the confidence threshold of 97% (Dudek *et al.* 2017). The obtained operational taxonomic units (OTUs) were then aligned using MUSCLE tool in CLC. The Alpha diversity indices (Simpson diversity index, Shannon diversity index, Chao1 index and Evenness index) were analysed and rarefaction curves describing the number of OTUs in all samples was generated using Paleontological Statistics Software Package for Education (PAST 3) software. The statistical significance was set at *p* < 0.05 for all indices. Estimation of differences in species diversity of microbes between localities was measured using Bray-Curtis distances for Beta diversity. UniFrac analysis then was conducted to describe the dissimilarity among *D. metesae* sample by considering both the evolutionary distances and the frequency of occurrences of bacterial phylotypes observed among samples (Phillips *et al.* 2012). The relation between localities was identified using principal coordinate analysis (PCoA) based on UniFrac metric and visualised using 3D graphics. A Venn diagram was generated to determine the shared and unique OTUs among localities at the 97% similarities. Phylogenetics dendrogram was constructed at genus level using Bray-Curtis distances with 1,000 bootstrap to define relationship between bacterial community populations of *D. metesae*. The correlation between the microbial diversity (genera) among the three localities was assessed using the Pearson correlation coefficient with *p* < 0.05. The correlation between localities was displayed by cold hot plot generated in PAST 3.

## RESULTS

**Figure 2:**
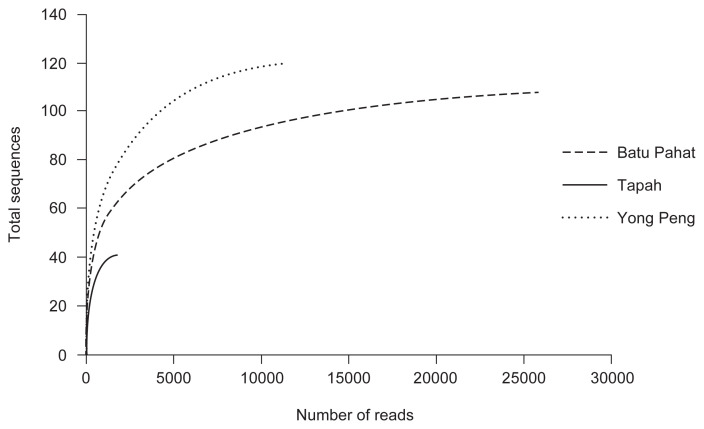
The rarefaction curve of the 16S rRNA gene sequences for different populations of *D. metesae* calculated for OTUs at 97% similarity.

A total of 39,198 bacterial 16S rRNA sequences were generated from three samples of *D. metesae*. The range of the assembled reads was 1,835 in Tapah sample to 25,976 in Batu Pahat sample (Table 2). Low-quality sequence reads were excluded, removal of chimeric sequences and clustering were done to obtain final data. At 97% similarity cut off, 170 OTUs were identified overall, ranging from 41 (Tapah) to 120 (Yong Peng) OTUs. Alpha diversity indices of OTUs observed in the populations of parasitoid wasp ranging from 1.764 to 2.844, 0.618 to 0.922, 41.86 to 121.8 for Shannon (H), Simpson 1-D and Chao1, respectively. The value of Shannon and Simpson diversity indices for *D. metesae* from Yong Peng shows the highest OTUs (*H* = 3.17, 1-D = 0.922), followed by *D. metesae* from Batu Pahat (*H* = 2.844, 1-D = 0.885) and *D. metesae* from Tapah (*H* = 1.764, 1-D = 0.61) (Table 2). The increasing rarefaction curve representing the number of OTUs, was not completely discovered or not yet observed by the number of sequences analysed. *D. metesae* from Tapah shows the lowest sequencing depth compared to other localities (Fig. 2).

**Figure 3:**
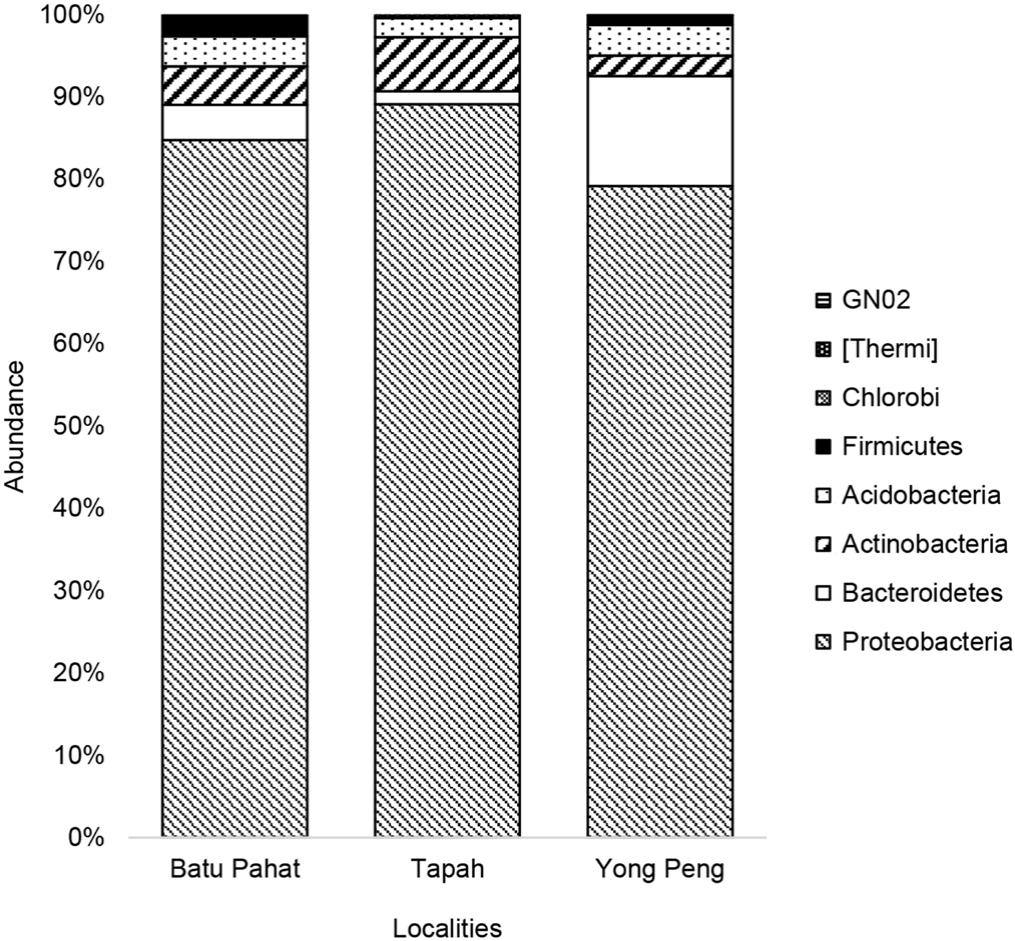
Relative abundances at the phylum level in parasitoid wasp, *D. metesae*.

**Figure 4:**
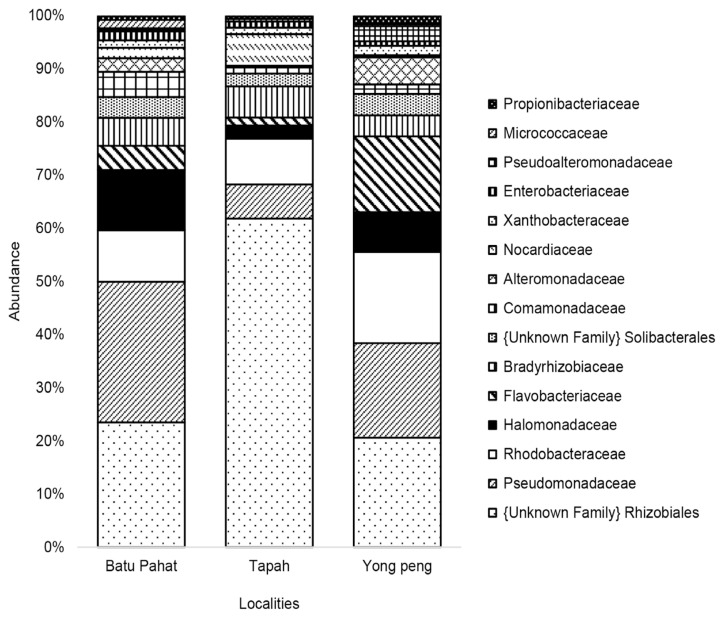
Relative abundances at the family level in parasitoid wasp, *D. metesae*.

**Figure 5:**
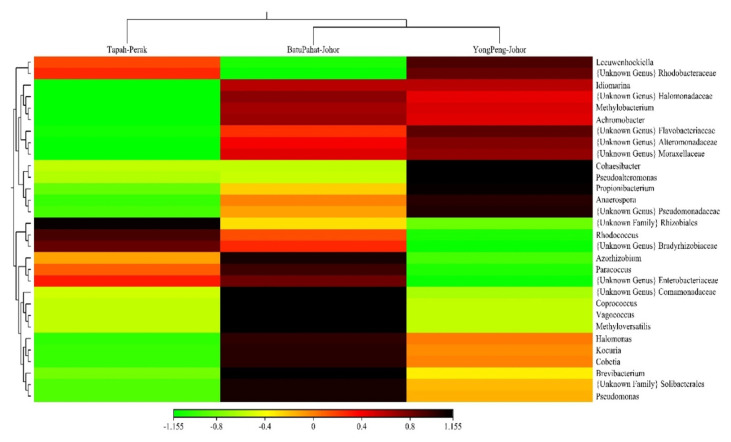
Heatmap with dendrogram at the genus level using a gradient heatmap (over 1% of the microbiome). The 30 most abundant genera were used in hierarchical clustering to evaluate the relationships between three samples of population of *D. metesae* using weighted pair clustering based on Bray-Curtis measurements. The darker colour indicate the more dominant the genus.

**Figure 6:**
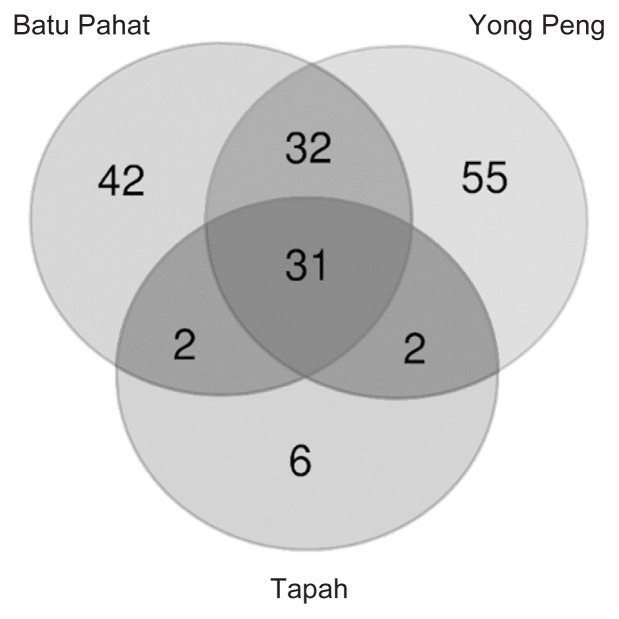
The Venn diagram illustrated the number of shared OTUs at the 97% similarity.

The OTUs were assigned to eight phyla, 54 families and 85 genera at 97% similarity. The eight phyla were consisting of Proteobacteria (83.31%), Bacteroidetes (6.80%), Actinobacteria (4.13%), Acidobacteria (3.60%), Firmicutes (2.11%), Chlorobi (0.02%), Thermi (0.02%) and unclassified bacteria (GNO2) (0.01%) (Table 3). Proteobacteria showed the highest abundance in all localities with 84.72%, 89.10% and 79.16% for Batu Pahat, Tapah and Yong Peng, respectively (Fig. 3). At the bacterial family level, 24.52% and 22.95% of 15 most dominant families were represented by an unknown family of order Rhizobiales and Pseudomonadaceae for all localities (Table 4) (Fig. 4). The heat map constructed shows 30 most dominant genera present in all localities where unknown genus of order Rhizobiales (23.36%) and *Pseudomonas* (21.20%) were the most dominant genera in all populations (Fig. 5). Out of total sequences, 31 OTUs were shared among populations in which *D. metesae* from Yong Peng has the most abundant OTUs with 55 unique sequences, followed by Batu Pahat with 42 unique sequences and Tapah with six unique sequences (Fig. 6).

**Figure 7:**
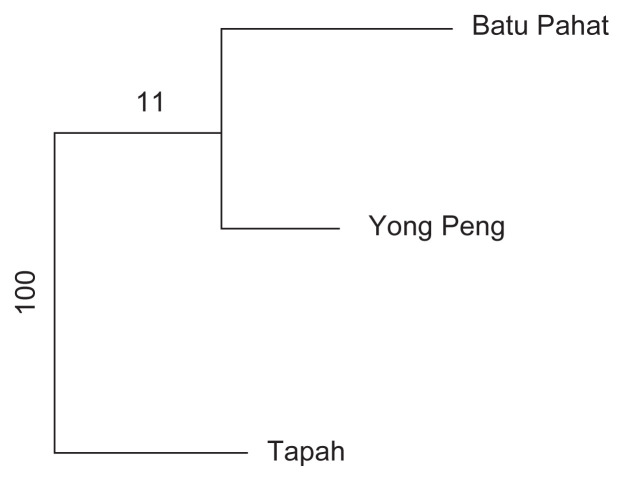
Phylogenetic tree dendrogram based on 16S rRNA using Bray-Curtis distance (at genus level).

**Figure 8:**
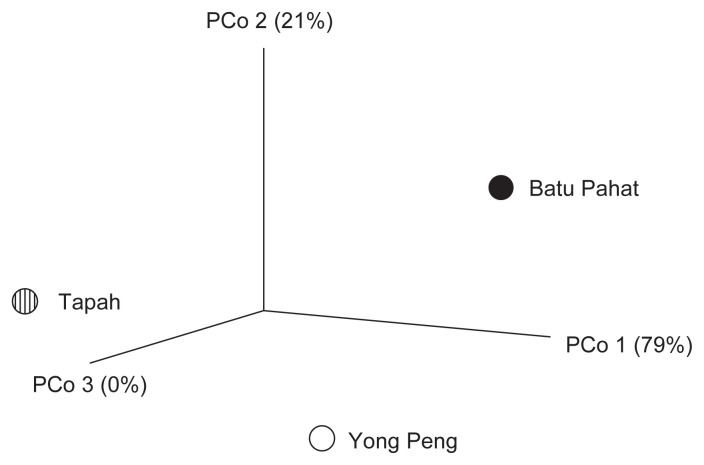
A three-dimensional plot of weighted UniFrac based principal coordinate analysis (PCoA). Plot was created using the pairwise weighted UniFrac distances (where PC1 is variability at 79%, PC2 is variability at 21%, and PC3 is variability at 0%).

**Figure 9:**
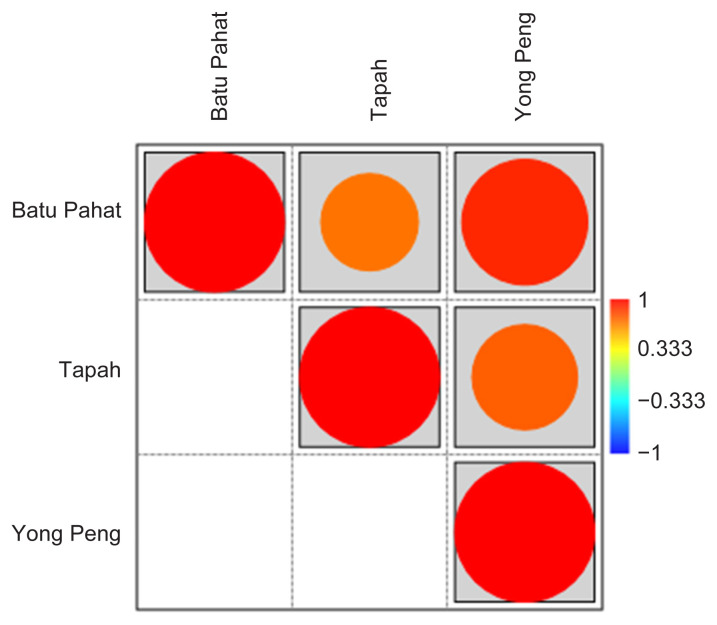
The cold-hot plot shows the correlation between the bacterial community (genera) between three population of *D. metesae*.

For Beta diversity, phylogenetic dendrogram was constructed based on Bray-Curtis distances where *D. metesae* from Batu Pahat and Yong Peng have close association of microbial community (Fig. 7). The result was supported by PCoA analysis based on UniFrac distance that shows a similar relationship pattern between the samples (Fig. 8). Through Pearson’s correlation analysis, the strongest correlation was detected from individuals of Batu Pahat and Yong Peng (*r* = 0.89827, *p* < 0.05), followed by *D. metesae* from Tapah and Yong Peng with *r* = 0.75358, *p* < 0.05 and samples from Batu Pahat and Tapah (*r* = 0.69552, *p* < 0.05), respectively (Table 5). Cold-hot plot shows the strength of the correlation relationships between localities ranging from 1 to −1 (Fig. 9).

## DISCUSSION

The culture techniques are limited to gain a complete picture of the microbes that occur naturally in the gut of *D. metesae.* These techniques are considered inefficient, time consuming and generate inadequate information (Hilton *et al.* 2016). Therefore, the metagenomic analysis using 16S rRNA was aimed to evaluate the uncultivated gut microbial diversity of the parasitoid wasp *D. metesae*. Our findings represent the first documentation of gut microbiome of *D. metesae*, a potential biological control agent in controlling *M. plana*, pest of oil palm in Malaysia.

The 16S rRNA gene is known as a highly conserved gene (Clarridge 2004) and is widely used in insects metagenomic analyses (Shi *et al.* 2013; Osimani *et al.* 2018; Pandiarajan & Krishnan 2018). The gene has also been commonly used in molecular phylogenetic studies as well as in bacterial taxonomy (Janda & Abbott 2007). Data generated from the 16S rRNA are important in ecological studies because the gut microbes allow the search for novel biocatalysts to develop innovative strategies in biotechnological applications (Krishnan *et al.* 2014). New proteins or enzymes that are selectively released by specific bacteria or other microbes through microbes-host association could be discovered (Heppel 1967), potentially leading to the development and commercialisation of novel biopesticides in controlling insect pests (Chattopadhyay *et al.* 2017). In addition, the study of parasitic wasp microbiomes can be significant in identifying those bacteria that were susceptible to insecticide applications to the pest species (Fernández *et al.* 2019).

This study extensively examined the microbial communities present in the gut of the parasitoid wasp, *D. metesae*, from three different localities of Peninsular Malaysia. Due to limited funding for this research, we decided to employ DNA pooling method as validated by Ray *et al.* (2019) that indicate its usefulness in sequencing microbiome at community level. The study revealed that the microbial diversity in our samples from Yong Peng and Batu Pahat (southern part) are closely related compared to the Tapah sample (northern part). This result was supported by the dendrogram based on Bray-Curtis distances, PCoA, number of shared OTUs and correlation analysis (Figs. 6–9, Table 5). This is also supported by the distance between locations which is the distance relatively shorter between Batu Pahat and Yong Peng (~28.2 km), whereas Tapah is located much further north of both Batu Pahat (~409.7 km) and Yong Peng (~385.4 km). Our data support the idea that species may have different microbiomes in different environments and geographical locations (Tasnim *et al.* 2017). In addition, different practices of IPM to control *M. plana* in the three locations (Batu Pahat, Yong Peng and Tapah) strongly support the results and may have a significant impact on the diversity of microbiome in the parasitic wasp, *D. metesae*. Indeed, our results show differences of the microbiome contents in *D. metesae* between Tapah (oil palm ecosystem with chemicals (cypermethrin and monocrothopos application) and Yong Peng–Batu Pahat (microbial biopesticide (*Bt*) and natural control), in line with Licht and Bahl (2018) who also reported chemical effects on bacterial communities. However, it is difficult to make conclusions from a sample size of three thus further studies with larger biological replicates and elimination of possible contaminants are recommended.

In this study, the analysis of microbial communities was done at phylum-level down to species level. Results showed that the predominant phyla found in the gut of *D. metesae* were Proteobacteria and Bacteroidetes with 83.31% and 6.80%, respectively. This result is supported by previous studies (Colman *et al.* 2012; Yun *et al.* 2014; Anusree Padmanabhan *et al.* 2019), in which Proteobacteria is one of the most dominant bacteria present. Proteobacteria was also reported as the predominant phylum in the smallest parasitoid wasp *Megaphragma amalphitanum* (Trichogrammatidae) (Nedoluzhko *et al.* 2017). In addition to Proteobacteria, phyla Actinobacteria and Bacteroidetes have also been commonly found in the gut of parasitic wasps (Fernández *et al.* 2019).

Interestingly, Rhizobiales fam. is the most abundant among 15 families recorded in this study, continued with Pseudomonadaceae. Rhizobiales fam. refers to an order of Gram negative Alphaproteobacteria, which is derived from the order Rickettsiales (Wooley & Ye 2010). The abundance of Rhizobiales fam. sp. was higher (61.81%) in the sample from Tapah, compared to the samples from Yong Peng (20.59%) and Batu Pahat (23.46%). However, the Pseudomonadaceae composition is found lower in Tapah (6.43%) compared to Yong Peng (17.80%) and Batu Pahat (26.44%). The composition of families also similar to the metagenomic result obtained from the mealybug species (Anusree Padmanabhan *et al.* 2019). Additionally, the family Micrococcaceae was absent in Tapah, but present in two other localities. The bacteria in the family of Micrococcaceae, e.g., from *Arthrobacter* and *Rothia* genera, also occur in the parasitic wasp subfamily Aphelinidae, but with low abundance (Fernández *et al.* 2019). It is hypothesised that high abundance of Rhizobiales fam. sp. and the absence of Micrococcaceae in the Tapah sample may be indirectly affected by their parasitoid host through IPM practices. In contrast, in Batu Pahat and Yong Peng no chemical applications are used in controlling *M. plana* populations. Natural control has been implemented in Batu Pahat and biopesticies (*Bt* application) in Yong Peng – do not appear to affect the parasitoid’s microbiome.

Based on the heat map, Rhizobiales gen. (23.36%) and *Pseudomonas* (21.20%) were the two top genera found in all populations. We believe that the composition really occurs in the gut of the wasps because we have minimising contamination by reducing up to 40% of the reads of PhiX using the Illumina MiniSeq to improve error in 16S sequences identification process (Jeon *et al.* 2015). There were several published papers indicated that Rhizobiales gen. occurred in the gut of insect by determination of genus *Candidiatus* in the cotton mealybug (*Phenacoccus solenopsis* Tinsley) on okra using metagenomic approach (Anusree Padmanabhan *et al.* 2019) and showed highest composition of genera. Several general bacteria species are known to have symbiotic interactions with their host by having a specific role, for example for host reproduction via feminisation and parthenogenesis in *Wolbachia* (Rickettsiales sister family of Rhizobiales) (Whitfield 2003; Mohammed *et al.* 2017), and also via thelytokous parthenogenesis in reproductive manipulation in *Rickettsia* (Gualtieri *et al.* 2017).

Paper by Anusree Padmanabhan *et al.* (2019) also indicated the presence of 0.03% of family Pseudomonadaceae in the mealybug gut. However, the low composition unable to detect the genus abundant in that species. *Pseudomonas* also has been recorded present in other insects such as tropical bed bug (Lim & Ab Majid 2021), mosquitoes and flies species (Sontowski & van Dam 2020). Despite advantageous in some cases of *Pseudomonas*’s infections, the function of this genus in *D. metesae* need to be investigated further.

## CONCLUSION

This study presents the first microbiome documentation of the parasitoid wasp *D. metesae*, as a natural biological control against the most dominant pests of oil palm, *M. plana*. In this study, Proteobacteria and Bacteroidetes were the two most abundant phyla in the gut of *D. metesae*. Interestingly, Rhizobiales fam. was the most abundant of the 15 families recorded in this study, while the family Micrococcaceae was absent in Tapah but present in the other two localities, suggesting that low diversity and richness in OTUs could be affected by the insecticide use in Tapah. The two most dominant genera were unknown genus of order Rhizobiales (23.36%) and *Pseudomonas* (21.20%) found in all populations. Low diversity and richness in Tapah might be due to direct and indirect effect of chemicals application in controlling the oil palm pests, *M. plana* which naturally parasitised by the *D. metesae.* This preliminary data represents a significant step towards the production of biopesticide by documenting of gut microbial of *D. metesae* that are susceptible to chemical insecticides, however thorough and many microbiome studies are required for these purposes.

## Figures and Tables

**Table 1:** List of samples used for microbiome analysis.

Sample code	Species	Locality	Practices
22	*D. metesae*	Batu Pahat, Johor	–
42	*D. metesae*	Yong Peng, Johor	Biopesticide, *Bt*
71	*D. metesae*	Tapah, Perak	Chemical (Cypermethrin and monocrothopos)

**Table 2:** Numbers of effective 16S rRNA gene sequences, numbers of observed OTUs, alpha diversity indices (Chao1, Shannon, and Simpson) and evenness for the gut bacterial community from three different localities.

Localities	No. of sequences	OTUs*	Chao1	Shannon (H)	Simpson (1-D)	Evenness (e)
Batu Pahat	25,976	107	107.30	2.844	0.885	0.1606
Tapah	1,835	41	41.86	1.764	0.618	0.1424
Yong Peng	11387	120	121.80	3.17	0.922	0.1984
Overall	39198	170				

*Note*:

* The operational taxonomical units (OTUs) were defined at the 97% similarity level.

**Table 3:** Relative abundance at the phylum level of microbiome communities in *D. metesae* from different localities.

No.	Phylum	Batu Pahat (%)	Tapah (%)	Yong Peng (%)
1	Proteobacteria	84.72	89.10	79.16
2	Bacteroidetes	4.29	1.58	13.36
3	Actinobacteria	4.69	6.54	2.48
4	Acidobacteria	3.63	2.34	3.74
5	Firmicutes	2.67	0.11	1.18
6	Chlorobi	0.00	0.00	0.07
7	Thermi	0.00	0.33	0.00
8	GN02	0.00	0.00	0.02

**Table 4:** Relative abundance at the family level of microbiome communities of *D. metesae*.

No.	Family	Batu Pahat (%)	Tapah (%)	Yong Peng (%)
1	{Unknown Family} Rhizobiales	23.46	61.81	20.59
2	Pseudomonadaceae	26.44	6.43	17.80
3	Rhodobacteraceae	9.73	8.59	17.14
4	Halomonadaceae	11.31	2.49	7.48
5	Flavobacteriaceae	4.56	1.50	14.27
6	Bradyrhizobiaceae	5.26	5.88	3.95
7	{Unknown Family} Solibacterales	3.90	2.38	4.02
8	Comamonadaceae	4.76	1.16	1.79
9	Alteromonadaceae	2.55	0.33	5.10
10	Nocardiaceae	1.96	5.93	0.39
11	Xanthobacteraceae	1.40	1.22	1.76
12	Enterobacteriaceae	1.69	1.16	0.86
13	Pseudoalteromonadaceae	0.59	0.50	2.86
14	Micrococcaceae*	1.56	0.00	0.40
15	Propionibacteriaceae	0.82	0.61	1.59

*Note*:

* Absence of Micrococcaceae in Tapah, Perak.

**Table 5:** The correlation coefficient values (Pearson *r*) and *p*-values (bold) of bacterial community (genera) among *D. metesae* of different localities.

	Batu Pahat	Tapah	Yong Peng
Batu Pahat		6.66E-26	6.97E-62
Tapah	**0.69552**		1.99E-32
Yong Peng	**0.89827**	**0.75358**	
